# Clinical Experience and Management Strategy of Retroperitoneal Tumor With Venous Tumor Thrombus Involvement

**DOI:** 10.3389/fonc.2022.873729

**Published:** 2022-05-10

**Authors:** Zhuo Liu, Liyuan Ge, Lei Liu, Xun Zhao, Kewei Chen, Yuxuan Li, Abudureyimujiang Aili, Min Lu, Xinlong Pei, Dengyang Han, Shudong Zhang, Lulin Ma

**Affiliations:** ^1^Department of Urology, Peking University Third Hospital, Beijing, China; ^2^Department of Radiation Oncology, Peking University Third Hospital, Haidian District, Beijing, China; ^3^Department of Pathology, School of Basic Medical Sciences, Peking University Third Hospital, Peking University Health Science Center, Beijing, China; ^4^Department of Radiology, Peking University Third Hospital, Beijing, China; ^5^Department of Anesthesiology, Peking University Third Hospital, Haidian District, Beijing, China

**Keywords:** retroperitoneal tumor, tumor thrombus, surgery strategy, postoperative outcome, clinical experience

## Abstract

**Background:**

This study aims to report the surgical management, complications, and outcomes for patients with retroperitoneal tumor and venous thrombus.

**Methods:**

We retrospectively analyzed 19 cases of retroperitoneal tumor with venous tumor thrombus from August 2015 to March 2021. A new tumor thrombus PUTH-RT grading system was proposed on the basis of the characteristics of the surgical techniques.

**Results:**

Two cases of PUTH-RT-1a, two cases of PUTH-RT-1b, six cases of PUTH-RT-2, six cases of PUTH-RT-3, and three cases of PUTH-RT-4 were included. Surgeries were successfully performed in all 19 patients. Among them, five cases (26.3%) were operated *via* a completely laparoscopic approach and 13 cases (68.4%) *via* an open approach. One case (5.3%) was converted from laparoscopic to open approach. Five cases (26.3%) experienced postoperative complications. All patients were followed for a median of 14 months. Cancer-associated death occurred in three cases. Distant metastases occurred in seven cases.

**Conclusions:**

We propose a new tumor thrombus grading system based on the anatomical characteristics of retroperitoneal tumors with venous tumor thrombus. Retroperitoneal tumor resection and removal of venous tumor thrombi are safe and effective for the treatment of such diseases.

## Introduction

Retroperitoneal tumors, such as adrenocortical carcinoma, adrenal pheochromocytoma, and retroperitoneal leiomyosarcoma, represent a group of anatomically similar tumors that occur in the retroperitoneum (1–3). Owing to the invasive nature of the tumors, these patients often present with venous system invasion at initial diagnosis (4, 5). Surgical extirpation is the cornerstone of treatment in these patients. Although radical nephrectomy and thrombectomy have been widely adopted in treating renal cell carcinoma with thrombus, the role of surgery in treating retroperitoneal tumors remains controversial, and preoperative surgical planning remains a challenge (6–9).

Open or laparoscopic surgery with en bloc excision has been the mainstay treatment for primary and recurrent retroperitoneal tumors because of the lack of effective adjuvant therapy (10–12). To date, there are no clear guidelines for the surgical treatment of retroperitoneal masses with venous involvement, partly due to the insufficient number of cases and the lack of prospective randomized trials (13). Owing to the rarity of this disease subset, it is crucial to summarize the surgical decision-making strategy and examine patients who undergo thrombectomy to describe their outcomes and complications.

We present a contemporary series of 19 patients diagnosed with retroperitoneal tumors with venous involvement, in which radical resection and thrombectomy were performed by means of open or laparoscopic surgery. On the basis of the characteristics of surgical techniques, we propose a new tumor thrombus management strategy stratification system and summarize its clinicopathological characteristics and prognosis to improve diagnosis and treatment experience.

## Materials and Methods

Following approval from the Peking University Third Hospital Ethics Committee Review Board, a retrospective analysis was conducted on 19 consecutive patients diagnosed with retroperitoneal tumors with venous thrombus from August 2015 to March 2021. The clinical presentation, imaging findings, extent and level of cava involvement, operative findings and technical details, estimated blood loss, postoperative complications, and overall survival were calculated.

According to the clinical manifestations, the patients were divided into those with no obvious clinical symptoms, those with only local symptoms (such as hematuria, lumbago, and abdominal mass), those with only systemic symptoms (such as fatigue, weight loss, and fever), and those with both local and systemic symptoms. Urinary system enhancing computed tomography (CT) scanning was performed to confirm the clinical diagnosis of retroperitoneal tumors and to determine whether hilar lymph node metastasis and distant metastasis of the abdominal organs were present. Enhanced magnetic resonance imaging (MRI) of the inferior vena cava was performed to identify the characteristics of the tumor thrombus. The length and width of the tumor thrombus in the inferior vena cava were measured in the coronal position. The presence or absence of thrombus (non-tumor emboli) was determined by the delayed period of MRI enhancement (14). On the basis of the imaging characteristics, whether the tumor thrombus had invaded the vessel wall was evaluated. A chest CT was performed to determine the presence of lung metastasis. For patients with bone pain and central nervous system symptoms, a bone scan and head MRI were performed to exclude bone metastasis and brain metastasis. When necessary, Positron Emission Tomography-Computed Tomography (PET-CT) was used to evaluate systemic metastasis. According to the imaging characteristics, whether the tumor thrombus invaded the vessel wall was evaluated. Chest CT was performed to determine whether there is lung metastasis. For patients with bone pain and central nervous system symptoms, bone scan and head MRI should be performed respectively, to exclude bone metastasis and brain metastasis. When necessary, PET-CT was used to evaluate systemic metastasis.

All patients underwent multidisciplinary treatment (MDT) discussions before the operation. The MDT team included urology, anesthesiology, radiology, ultrasound diagnosis, oncology, pathology, general surgery, and cardiac surgery. The MDT clinical treatment decision was based on comprehensive opinions from various disciplines, including preoperative preparation, surgical methods, and coping strategies for special conditions during operation, prevention, and treatment strategies for postoperative complications.

The patients were grouped according to the surgical approach as follows: complete laparoscopic approach, open approach, and laparoscope-to-open approach. We have previously described the incision, body position, and puncture location of different surgical approaches (15–17).

**Figure 1:**
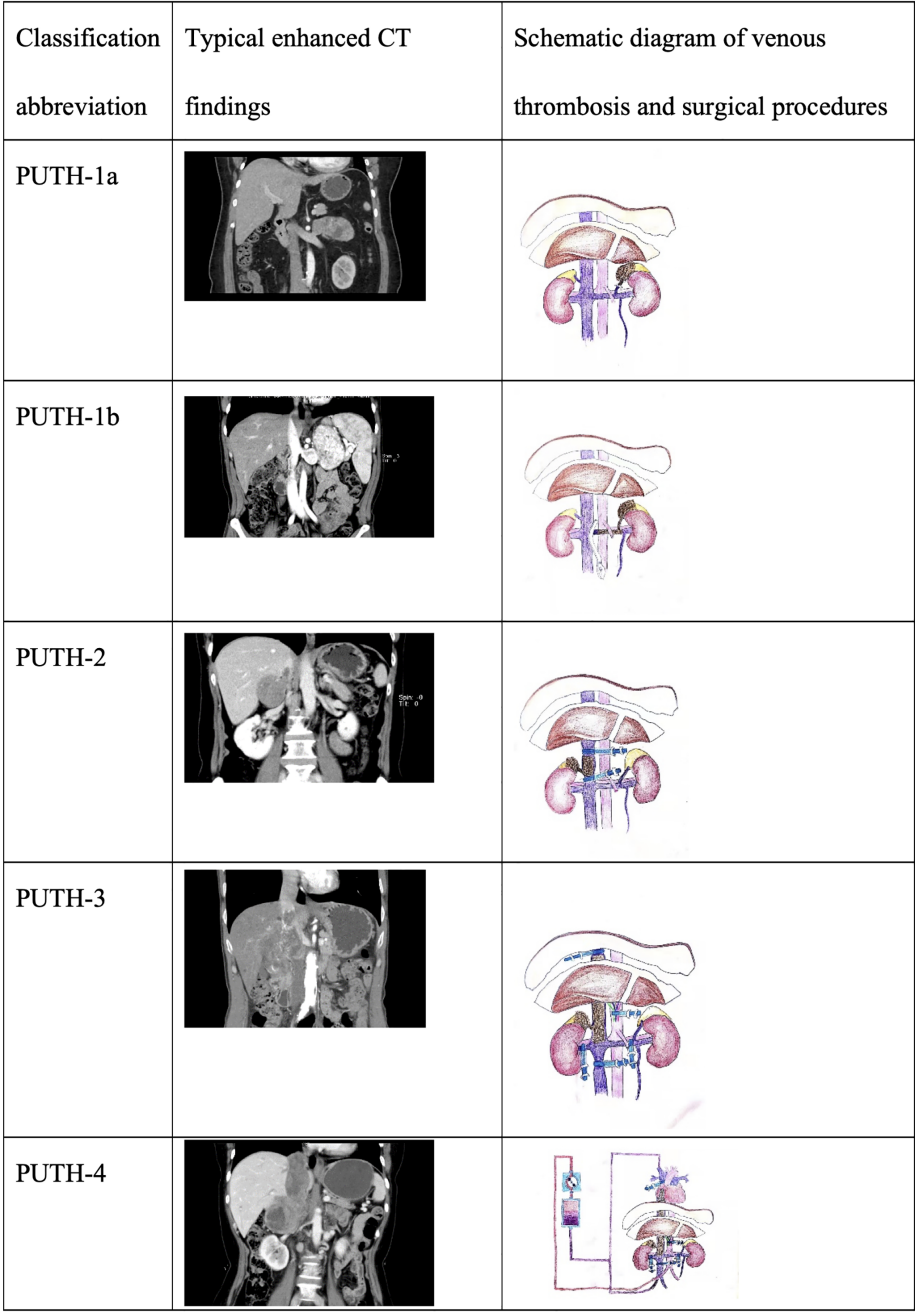
Typical imaging manifestations and schematic diagram of PUTH-RT grading system for adrenal gland with venous thrombosis.

First, we used the Mayo grading system, a classical grading method for renal cell carcinoma and venous tumor thrombus, to evaluate patients with retroperitoneal tumors with venous tumor thrombus (18). Simultaneously, we classified all cases using the Peking University Third Hospital (PUTH-RT) grading system (**Figure 1**) based on the PUTH-RT grading system reclassification (**Table 1**). For PUTH-RT 1a grade tumor thrombus in the left adrenal gland, the left central adrenal vein can be cut off with a Hem-o-lok clamp or a straight-line cutter. For a PUTH-RT 1b grade tumor thrombus in the left adrenal gland, Satinsky forceps can be used to partially block the inferior vena cava. The left renal vein was then cut during the operation, the tumor thrombus of the left adrenal gland and the central adrenal vein were removed, and the left renal vein was sutured to remove the blockage. If a tumor thrombus invades the wall of the left renal vein, then a Hem-o-lok clamp or straight-line cutter is used to cut off the left renal vein. The left kidney circulates only through the left gonadal vein or collateral branch of the ascending lumbar vein. For adrenal PUTH-RT grade 2 tumor thrombi, the tumor thrombus enters the inferior vena cava, the proximal end of the tumor thrombus is located under the liver, and the distal end of the tumor thrombus is located above the level of the renal vein. During the operation, the inferior vena cava at the distal end of the tumor thrombus and the inferior vena cava at the proximal end of the tumor thrombus can be blocked by vascular blocking tape, and the inferior vena cava can be cut open to remove the thrombus. If the distal end of the tumor thrombus is born in the direction of blood flow, then it can be located below the level of the renal vein. During the operation, blood vessel blocking tape was used to block the inferior vena cava of the distal and proximal ends of the tumor thrombus; it was also necessary to block the blood reflux of the left renal vein. For PUTH-RT grade 3 tumor thrombi of the adrenal gland, the proximal end of the tumor thrombi reached the level of the retroliver. During the operation, it is necessary to use the technique of liver turning to break off the ligament and expose and free the inferior vena cava behind the liver. The first hepatic portal vein was dissociated using the Pringer method (19). The inferior vena cava, first hepatic portal vein, left renal vein, and inferior vena cava near the heart end of the tumor thrombus were blocked by the vascular blocking belt, and then, the inferior vena cava was cut to remove the thrombus. For PUTH-RT 4 grade tumor thrombus of the right adrenal gland, we can first try to open the diaphragm without thoracotomy, that is, cutting the diaphragm central tendon around the vena cava or cutting the diaphragm directly. The tumor thrombus was squeezed gently into the inferior vena cava, so that the tumor thrombus changed from above the diaphragm to below the diaphragm, before removing the tumor thrombus. Alternatively, the chest can be opened to establish cardiopulmonary bypass. Incision of the right atrium is performed to remove the thrombus under hypothermic cardiac arrest or non-cardiac arrest. The modified Clavien grading system was used to evaluate postoperative complications (20). Grade III or higher complications were defined as severe. We performed hilar or retroperitoneal lymphadenectomy for patients with enlarged renal hilar or retroperitoneal lymph nodes on preoperative imaging or for those in whom enlarged lymph nodes found during surgery.

**Table 1:** PUTH-RT grading system of adrenal tumor thrombus with vein based on surgical technique.

Classification Abbreviation	Graded Full Name	Description	Case Load	Description of Surgical Technique
PUTH-RT-1a	Adrenal PUTH-RT 1a grade tumor thrombus	Cancer thrombus is confined to the central adrenal vein.	2	Hem-o-lok was used to clamp or cut off the central adrenal vein.
PUTH-RT-1b	Adrenal PUTH-RT 1b grade tumor thrombus	The tumor thrombus entered the left renal vein along the left central adrenal vein but did not enter the inferior vena cava.	2	During the operation, the inferior vena cava was partially blocked by side-wall forceps, and the left renal vein was incised during the operation. After taking out the tumor thrombus, the left renal vein was sutured. If tumor thrombus invades the wall of left renal vein, then Hem-o-lok clamp or straight-line cutter should be used to cut off the left renal vein.
PUTH-RT-2	Adrenal PUTH-RT grade 2 tumor thrombus	The tumor thrombus invades the inferior vena cava, and its proximal end is located under the liver.	6	If the distal end of the tumor thrombus is located above the level of renal vein, then the vena cava of the distal end of the tumor thrombus is blocked by the vascular blocking tape in turn, and the inferior vena cava is cut after the tumor thrombus is close to the vena cava. If the distal end of the tumor thrombus is below the level of renal vein, vascular occlusion needs to block the blood reflux of the healthy renal vein at the same time.
PUTH-RT-3	Adrenal PUTH-RT grade 3 tumor thrombus	The tumor thrombus invades the inferior vena cava, and its proximal end is located behind the liver.	6	During the operation, the liver ligament was severed, the inferior vena cava behind the liver was exposed and dissociated, and the first hepatic portal was dissociated by Pringer method. The distal vena cava, the first hepatic portal vein, the contralateral renal vein, and the proximal vena cava of the tumor thrombus were blocked, in turn, by the vascular blocking band, and then, the inferior vena cava was cut to remove the thrombus.
PUTH-RT-4	Adrenal PUTH-RT grade 4 tumor thrombus	The tumor thrombus invaded the inferior vena cava and reached above the diaphragm.	3	You can cut the diaphragm central tendon around the vena cava or cut the diaphragm directly, gently push, and squeeze the tumor thrombus into the inferior vena cava, so that the tumor thrombus changes from above the diaphragm to below the diaphragm and then take out the tumor thrombus; conventional methods need to open the chest to establish cardiopulmonary bypass and open the right atrium to remove thrombus under the condition of hypothermic cardiac arrest or non-cardiac arrest.

The first follow-up was conducted within 1 month of the operation, focusing on postoperative complications. Patient follow-up was performed every 3–6 months for the first 2 years and every 6–12 months thereafter for 5 years. A tyrosine kinase inhibitor was administered to patients with residual tumor during surgery, recurrence, or distant metastasis after surgery. The curative effects and adverse reactions of the drug were evaluated.

## Results

### Patient Demographics

Nineteen patients with retroperitoneal tumors who underwent radical resection and thrombectomy between August 2015 and March 2021 were included in this study. The patients’ data, including age, sex, and tumor burden, are listed in **Tables 2**, **3**. The median age of all patients at the time of surgical treatment was 42 years (range, 16–69 years). Among the 19 patients, there were 7 males and 12 females. The body mass index (BMI) ranged from 18.3 to 36.2 kg/m^2^, and the median BMI was 24.1 kg/m^2^. Retroperitoneal tumors were located on the right side in 13 patients and on the left side in six patients. The median tumor diameter was 9.1 cm (range, 2.8–18.3 cm). The median tumor thrombus length and width were 4 cm (1.0–12.3 cm) and 2.3 cm (1.0–3.9 cm), respectively.

**Table 2:** Baseline characteristics of all 19 patients.

Characteristics	Result
Age (years), median	42 (16–69)
Gender, male/female	7/12
BMI (kg/m2), median	24.1 (18.3–36.2)
Side, n	
Left	6
Right	13
Tumor size (cm), median	9.1 (2.8–18.3)
Clinical N stage, n	
cN0	13
cN1	6
Clinical M stage, n	
cM0	13
cM1	6
IVC thrombus length (cm), mean (SD)	4.0 (1.0–12.3)
Clinical symptoms, n	8
No	8
Local symptoms	3
Systemic symptoms	

BMI, body mass index; IVC, inferior vena cava; SD, standard deviation.

Data presented as mean (SD).

**Table 3:** Clinical characteristics of patients in our cohort (n = 19).

ID	Pathology	PUTH-RTGrade	Mayo	Gender	Age	BMI (kg/m^2^)	Symptoms	Side	Diameter(cm)	ASAGrade	cN stage	cMstage	Combined thrombi	ThrombusLength(cm)	ThrombusWidth(cm)
1	ccRCC (metastasis)	PUTH-RT 4	IV	F	68	23.8	Systemic	R	6.3	3	cN1	M1	N	5.5	2.7
2	Leiomyosarcoma (primary site)	PUTH-RT 2	II	M	31	20.3	Local	R	9.0	2	cN1	M0	Y	4.0	2.9
3	PHEO	PUTH-RT 3	II	M	44	21.0	Local	R	10.0	3	cN0	M0	Y	4.7	3.0
4	ACC	PUTH-RT 3	III	F	31	30.4	Local	R	15.0	2	cN0	M0	N	5.0	2.1
5	hepatocellular carcinoma (metastasis)	PUTH-RT 2	II	F	67	28.9	Local	L	2.8	2	cN1	M1	N	5.9	2.7
6	Adrenal adenoma	PUTH-RT 1a	0	F	42	33.7	No	L	7.9	2	cN0	M0	N	1.3	1.2
7	Ewing Sarcoma (primary site)	PUTH-RT 4	IV	F	20	18.6	Local	R	18.3	4	cN0	M1	N	12.3	3.0
8	ACC	PUTH-RT 1a	0	M	58	24.1	No	R	9.4	2	cN0	M0	N	1.5	1.0
9	Leiomyosarcoma (primary site)	PUTH-RT 3	III	M	55	18.3	No	R	10.7	2	cN0	M0	N	7.5	3.2
10	Paraganglioma	PUTH-RT 2	I	M	16	21.0	Systemic	R	9.1	1	cN0	M0	N	1.0	1.0
11	Leiomyosarcoma (primary site)	PUTH-RT 4	IV	F	57	24.7	Local	R	7.1	3	cN1	M1	N	12.0	3.9
12	Leiomyosarcoma (primary site)	PUTH-RT 3	II	F	56	28.7	No	R	5.8	2	cN0	M1	N	2.8	1.6
13	ACC	PUTH-RT 2	II	F	56	36.2	No	R	6.5	3	cN0	M0	N	4.0	1.0
14	PHEO	PUTH-RT 1b	0	F	35	21.5	No	L	7.4	2	cN0	M0	N	2.3	1.3
15	ACC	PUTH-RT 2	II	F	49	21.5	Local	L	12.2	2	cN0	M0	N	4.0	2.3
16	ACC	PUTH-RT 3	II	F	69	23.1	Systemic	R	12.5	2	cN1	M1	N	3.0	2.3
17	ACC	PUTH-RT 2	II	F	54	26.0	No	R	7.1	2	cN0	M0	N	3.3	1.4
18	Adrenal neuroblastoma	PUTH-RT 3	II	M	38	25.1	Local	L	10.3	2	cN1	M0	N	7.0	3.2
19	PHEO	PUTH-RT 1b	0	M	53	24.2	No	L	8.8	2	cN0	M0	N	2.5	2.1

ccRCC, clear cell renal cell carcinoma; PHEO, pheochromocytoma; ACC, adrenal cortical carcinoma.

We classified the patients into five grades according to the PUTH-RT grading system based on the location of the retroperitoneal tumors and thrombus level. The five grades were defined as follows: PUTH-RT-1a, tumor thrombus confined in the central adrenal vein; PUTH-RT-1b, a left-side retroperitoneal tumor with thrombus extending into the left renal vein along the left central adrenal vein but not reaching the inferior vena cava; PUTH-RT-2, tumor thrombus invading the inferior vena cava, with the proximal end located under the liver; PUTH-RT-3, tumor thrombus invading the inferior vena cava, with the proximal end located behind the liver; and PUTH-RT-4, tumor thrombus invading the inferior vena cava and reaching above the diaphragm. The preoperative clinical data of the 19 patients are presented in **Table 4**.

**Table 4:** Perioperative data and pathologic result of 19 patients.

Characteristics	Result
Operative time (min), median (IQR)	342 (153–575)
Estimated blood loss (ml), median (IQR)	1,300 (200–4,500)
Patients receiving transfusion, n (%)	14 (73.7)
Postoperative hospital stay (d), mean (SD)	8 (4–44)
ASA grade, n (%)	
Grade I	1 (5.3)
Grade II	13 (68.4)
Grade III	4 (21.1)
Grade IV	1 (5.3)
Postoperative complication, n (%)	5 (26.3)
Preoperative serum Cr (μmol/L), median (IQR)	68 (48–90)
Postoperative (1 week) serum Cr (μmol/L), median (IQR)	66 (41–129)
IVC wall invasion, n (%)	6 (31.6)
Presence of bland thrombus, n (%)	2 (10.5)
Lymph node metastasis, n (%)	3 (15.8)

IQR, interquartile range; SD, standard deviation; ASA, American Society of Anesthesiologists; Hb, hemoglobin; Cr, creatinine; BUN, blood urea nitrogen; ccRCC, clear cell renal cell carcinoma; IVC, inferior vena cava. Data presented as mean (SD) or median (IQR), unless otherwise noted.

### Intra-Operative Outcomes

Among the included patients, five (26.3%) were operated *via* a completely laparoscopic approach and 13 (68.4%) were operated *via* an open approach. One patient underwent laparoscopic conversion to open surgery, which was planned before the surgery. First, the retroperitoneal tumor and kidney were dissociated using the laparoscopic approach, and then the inferior vena cava tumor thrombus was resected by open surgery. This laparoscopic-to-open conversion approach combines the advantages of minimally invasive laparoscopic treatment with the safety of open surgery. The retroperitoneal laparoscopic approach has advantages for ligation of the renal artery. During the treatment of a tumor thrombus, the open approach has the advantages of a larger operation space and in staunching bleeding. We have described our experience in managing renal tumors with inferior vena cava tumor thrombus in previous studies, and we used this active transfer to an open method in managing retroperitoneal tumors with tumor thrombus (16). Eighteen patients underwent radical resection of the retroperitoneal tumor and venous tumor thrombectomy, and one patient underwent palliative retroperitoneal tumor resection due to complete adhesion between the thrombus and vessel wall. Of the 19 patients, 13 patients had the ipsilateral kidney preserved during the operation, and six patients had it removed. The median operative time was 342 min (range, 153–575 min). The median intraoperative blood loss was 1,300 ml (range, 200–4,500 ml). Fourteen of the 19 patients (73.7%) received intraoperative blood transfusions, with a median transfusion requirement of 4 units (range, 2–6 units) (**Table 5**).

**Table 5:** Operation data of patients in our cohort (n = 19).

ID	Approach	Ipsilateral nephrectomy	Time	Blood loss	RCS transfusion	Plasma transfusion	LN resection	Segmental IVC resection
1	Lap to O	Y	526	2,000	800	0	Y	N
2	O	Y	288	400	0	0	N	N
3	O	N	486	2,700	1,600	800	N	N
4	O	N	504	3,500	3,200	1,400	N	N
5	O	N	216	1,500	1,200	0	N	N
6	Lap	N	153	200	0	0	N	N
7	O	N	587	4,500	2,800	2,200	N	N
8	Lap	N	193	200	0	0	N	N
9	O	Y	256	800	800	0	N	N
10	O	Y	329	1,000	1,200	400	N	N
11	O	N	297	2,000	2,800	800	N	Y
12	O	N	377	900	600	0	N	N
13	Lap	N	335	800	400	0	N	N
14	Lap	N	217	100	0	0	Y	N
15	O	Y	440	2,000	1,600	400	Y	N
16	O	N	355	3,500	2,800	1,400	Y	N
17	O	N	261	900	0	0	N	N
18	O	Y	575	3300	2800	1600	Y	N
19	Lap	N	429	1600	800	400	N	N

Lap to O, laparoscopic approach to open approach; O, open approach; Lap, laparoscopic approach.

### Surgical Outcomes

Among the 19 patients, 12 tumors originated from adrenal tissue, including six adrenal cortical carcinomas, three pheochromocytomas, one paraganglioma, one adrenal adenoma, and one adrenal neuroblastoma. There were seven cases of other retroperitoneal tumors not from adrenal tissue, including four leiomyosarcomas (primary site), one clear cell carcinoma of the kidney (adrenal metastasis), one hepatocellular carcinoma (adrenal metastasis), and one Ewing sarcoma (primary site). The pathological type was unclear in one case. Of 19 patients, six (31.6%) had vessel wall invasion, two (10.5%) had inferior vena cava thrombosis (non-tumor thrombus), and three (15.8%) had lymph node metastasis.

No perioperative deaths occurred during the first 30 days after the surgery. Postoperative complications occurred in five patients (26.3%); among whom, there were three cases of anemia (Clavien grade 2), one case of wound infection (Clavien grade 1), and one case of pulmonary embolism (Clavien grade 2). Three patients recovered from postoperative anemia after red blood cell suspension transfusion. The patient with wound infection had delayed union after two weeks of wound dressing. The patient with a pulmonary embolism recovered after treatment with low–molecular weight heparin. The median postoperative hospital stay was 8 days (**Table 6**).

**Table 6:** Postoperative data of patients in our cohort (n = 19).

ID	P.O.stay	Pre-Cr (μmol/L)	Post-Cr (μmol/L)	pN stage	IVC invasion	Complication	Follow-up	Status	Recurrence	P.O. treatment
1	8	54	53	Y	N	Infection	69.0	Alive	Lung	Sunitinib
2	8	88	129	N	Y	–	49.0	Alive	No	–
3	10	81	66	N	Y	Anemia	41.0	Alive	No	–
4	9	63	66	N	N	Anemia	10.0	Alive	Liver metastasis	Radiotherapy
5	8	67	87	N	N.A	–	7.0	Alive	No	–
6	4	48	54	N	N	–	3.0	Alive	Lymph node	Chemotherapy
7	44	67	118	N	N	Anemia	21.0	Alive	No	–
8	5	65	57	N	N	–	5.0	Alive	No	–
9	6	74	93	N	N	–	27.0	Alive	No	–
10	8	90	81	N	Y	–	26.0	Alive	No	–
11	16	78	64	N	Y	–	7.0	Dead	No	–
12	13	68	41	N	N	P.E.	24.0	Alive	No	–
13	8	54	58	N	N	–	7.0	Alive	Multiple organ	–
14	5	60	87	N	N	–	22.0	Alive	No	–
15	9	56	45	N	Y	–	16.0	Alive	–	–
16	10	71	50	N	N	–	11.0	Dead	Liver	–
17	5	75	88	N	N	–	14.0	Dead	Retroperitoneal	Immnotherapy
18	12	90	103	N	Y	–	9.0	Alive	Diaphragmatic	Erlotinib
19	4	71	93	N	N	–	3.0	Alive	No	–

P.O., postoperative; P.E., pulmonary embolism.

## Discussion

At present, most research concerning venous thrombi focuses on renal cell carcinoma. However, there are few studies on adrenal tumors or other retroperitoneal tumors with venous tumor thrombi (13, 21). The Mayo thrombus grading system is commonly used for the evaluation of renal cell carcinoma (18). The Mayo classification mainly considers the level of tumor thrombus, and different Mayo grades correspond to different surgical procedures, difficulties, and complications. Some studies have suggested that the higher the Mayo grade, the worse the prognosis of patients. However, the Mayo grading is mainly applied to renal cell carcinoma with inferior vena cava tumor thrombi. Retroperitoneal tumors with venous tumor thrombus, represented by adrenal tumors, differ from classical renal cell carcinoma with venous tumor thrombus in terms of their anatomical structure and characteristics. First, the kidney tumor is located inside the kidney, whereas the adrenal tumor is located at the upper pole of the kidney, between the liver and kidney, or between the spleen and kidney. This indicates that the proximal end of a venous thrombus is often higher. The operation is complex due to the dissociation of the tumor thrombus near the proximal end. In this study, according to the traditional Mayo classification method, 10 patients were classified as Mayo II; however, the liver still needed to be freed and the liver-related ligaments cut off to expose the posterior inferior vena cava. In this case, it is necessary to block the first hepatic portal vessel to reduce bleeding. These procedures are usually applied to patients with a Mayo grade III tumor thrombus in renal cell carcinoma venous tumor thrombus. Second, the adrenal tumors were closer to the liver or spleen. Right adrenal tumors often adhere to or invade the liver, which can easily cause bleeding during surgery and increase the difficulty of the operation, posing a significant challenge for the safety of surgery. Third, there are great differences in the anatomical characteristics between the central adrenal vein and the renal vein. In renal cell carcinoma, the tumor grows from the kidney into the renal vein and then directly enters the inferior vena cava in the blood reflux direction, which is different for adrenal tumors. There were also significant differences between left adrenal tumors and right adrenal tumors. Right adrenal tumors can enter the inferior vena cava after entering the right central adrenal vein. The left adrenal tumor first enters the left renal vein and then grows along the left renal vein into the inferior vena cava.

Therefore, there is a considerable difference between surgical treatment of adrenal tumors with venous thrombosis and that of traditional renal cell carcinoma with venous thrombosis. At present, there is no corresponding grading system for adrenal or retroperitoneal tumors with venous tumor thrombi. In this study, we propose the use of a PUTH-RT scoring system. In PUTH-RT 1 grade, the patients were further divided into stages 1a and 1b according to the different sides and relationships with the renal vein. Among these, stage 1b is the most suitable for patients with a left adrenal tumor thrombus. The tumor thrombus enters the left renal vein along the left central adrenal vein but not the inferior vena cava. Satinsky forceps can be used to partially block the inferior vena cava, cut the left renal vein, remove the left adrenal tumor and the tumor thrombus in the central adrenal vein, suture the left renal vein, and then remove the blockage. If the tumor thrombus does not invade the vein wall of the left kidney, then the left kidney can be preserved. However, for a tumor thrombus invading the left renal vein wall, a Hem-o-lok clamp or straight-line cutter should be used to cut off the left renal vein. The left kidney depends on collateral circulation, such as *via* the gonad vein and lumbar ascending vein, for reflux. If an adrenal tumor thrombus extensively invades the left renal vein, then simultaneous resection of the left kidney is required to ensure radical resection of the tumor, even if the left renal vein is unaffected. The treatment method for PUTH-RT grades 2 to 4 is similar to that for Mayo grades II to IV. The difference lies in the different positions of the distal end of the tumor thrombus and surgical procedures. If the distal end of the tumor thrombus is above the level of the renal vein, then it is necessary to block the distal vena cava of the cancer thrombus and the proximal vena cava of the cancer thrombus using a vascular blocking belt and then cut the inferior vena cava to remove the thrombus. If the distal end of the tumor thrombus is below the level of the renal vein, then vascular occlusion must simultaneously block blood reflux from the healthy renal vein.

In terms of the surgical approach, 14 patients (70%) underwent the open approach. At our center, we selected the surgical approach by referring to the PKUTHLP score, as reported previously (21). Briefly, the clinical lymph node stage (without lymph node invasion = 0, with lymph node invasion = 1), Mayo classification (Mayo 0–1 = 0, Mayo 2 = 2, Mayo 3–4 = 3), and tumor diameter (size < 10 cm = 0, size ≥ 10 cm = 1) were used to construct the PKUTHLP score system, which ranges from to 0–5. For PKUTHLP score > 2, we prefer to perform open surgery; otherwise, we select laparoscopic surgery. With the wide application of minimally invasive technology, many low-grade cancer thrombectomies can be completed using a complete laparoscopic or robotic approach; however, the open approach is still an irreplaceable traditional and effective method for treating tumor thrombi (22). The proportion of open approach surgeries is very high in adrenal tumors associated with venous tumor thrombi. This is largely determined by operating space. Because of the ipsilateral kidney, the space is extremely narrow, which constrains the application of minimally invasive techniques in adrenal tumors with venous tumor thrombus. In addition, because adrenal tumors are located in the upper pole of the kidney, the proximal end of the venous tumor thrombus is often higher. To fully expose the proximal end of the cancer thrombus, it is necessary to free the liver during the operation, but these operations are technically difficult using a minimally invasive approach. Moreover, the laparoscopic approach may increase the risk of tumor implantation and diffusion due to tumor rupture. We believe that retroperitoneal tumor and inferior vena cava tumor thrombus removal are effective treatment options despite the risk of surgical complications. In this study, five patients experienced postoperative complications, including one case of pulmonary embolism. This type of operation is complex, and the operator should fully explain the benefits and potential risks of the operation to patients and their families before the operation.

Regarding whether the ipsilateral kidney should be removed from adrenal tumors, we aim to preserve the ipsilateral kidney as much as possible. If one of the following conditions occurs, then resection of the ipsilateral kidney can be considered: 1. the adrenal tumor is strongly adhered to the ipsilateral kidney, or the tumor surrounds the hilar blood vessel and cannot be separated; 2. preoperative imaging suggests that the adrenal tumors invaded the ipsilateral kidney; and 3. the tumor thrombus extensively invades the blood vessel wall of the ipsilateral renal vein, and collateral venous circulation cannot be compensated.

Adrenal tumors with venous tumor thrombus are often of high grade, and transesophageal ultrasound plays a critical role in monitoring the thrombus during surgery (23). After anesthesia induction, patients can be examined using transesophageal ultrasonography before, during, and after thrombectomy. Before thrombectomy, vena cava wall invasion can be determined by monitoring the blood flow between the tumor thrombus and vessel wall. During thrombectomy, transesophageal ultrasonography can evaluate whether the inferior vena cava vascular occlusion band is placed above the proximal end of the tumor thrombus and dynamically observe the entire process of tumor thrombectomy. After resection of the tumor thrombus, transesophageal ultrasonography was performed to observe the presence of a residual tumor thrombus in the vena cava. Compared to the “static” and “past” information of preoperative imaging examination, such as CT or MRI, the real-time and dynamic monitoring of heart, inferior vena cava, and intraluminal tumor thrombus by transesophageal ultrasound can further clarify and even correct the preoperative diagnosis. It is crucial to provide real-time information of tumor thrombus for surgical decision-making and operation.

This study has several limitations. First, the PUTH-RT grading system described in this study is suitable for the common adrenal vein anatomy. However, venous variations may occur in a few patients, which requires surgeons to read me carefully before the operation and make individualized surgical plans according to the specific characteristics of each patient. Second, the sample size in this group was relatively small. This is mainly due to the low incidence of venous thrombosis in adrenal or retroperitoneal tumors. A multicenter study with a larger sample size is required for further verification. Third, the main classification basis of the adrenal tumor venous thrombus system in this study was determined according to the anatomical position of the tumor thrombus level. The pathological types of retroperitoneal tumors included pheochromocytoma, paraganglioma, adrenal adenoma, adrenal neuroblastoma, renal clear cell carcinoma, hepatocellular carcinoma, and Ewing sarcoma. As the above tumors are in the retroperitoneum and accompanied by inferior vena cava tumor thrombus, there are some similarities in the surgical treatment methods. We classified all retroperitoneal tumors into a research group. Owing to the large heterogeneity of tumor pathological types, this may weaken the reliability of survival results. Here, we report the survival results on a case-by-case basis. The purpose of this study was to introduce the surgical treatment of retroperitoneal tumors (not purely of renal origin) with inferior vena cava tumor thrombus. In this study, the description and schematic diagram of our grading system consider adrenal tumors as an example in an attempt to intuitively reflect the disease characteristics and surgical treatment points for easy understanding. However, not all of the subjects in this study are from adrenal gland, which may be slightly different in the description and schematic diagram of the grading system. The paraganglioma described in this study is a retroperitoneal tumor and is not from adrenal tissue, because ipsilateral normal adrenal tissue can be observed in imaging examination.

## Conclusions

In summary, in this study, we proposed a new classification system for tumor thrombus based on the anatomical characteristics of adrenal tumors or other retroperitoneal tumors associated with venous thrombosis. Retroperitoneal tumor resection and venous tumor thrombectomy are safe and effective treatments for these diseases.

## Data Availability Statement

The original contributions presented in the study are included in the article/supplementary material. Further inquiries can be directed to the corresponding authors.

## Ethics Statement

This study was reviewed and approved by the Peking University Third Hospital Ethic Committee (protocol code 2021-245-02 and date of approval May 29, 2021) and was conducted according to the guidelines of the Declaration of Helsinki. Written informed consent was obtained from all participants for their participation in this study.

## Author Contributions

Conceptualization: ZL and LG; methodology: LL, KC, and YL; validation: XZ and AA; formal analysis: XP and KC; investigation: XZ; resources: ML; data curation: DH; writing—original draft preparation: ZL and LG; writing—review and editing: LM; visualization: SZ. All authors contributed to the article and approved the submitted version.

## Conflict of Interest

The authors declare that the research was conducted in the absence of any commercial or financial relationships that could be construed as a potential conflict of interest.

## Publisher’s Note

All claims expressed in this article are solely those of the authors and do not necessarily represent those of their affiliated organizations, or those of the publisher, the editors and the reviewers. Any product that may be evaluated in this article, or claim that may be made by its manufacturer, is not guaranteed or endorsed by the publisher.
